# Assisting people with dementia with their medicines: experiences of family carers

**DOI:** 10.1111/ijpp.12158

**Published:** 2014-10-28

**Authors:** Felicity Smith, Madelon S Grijseels, Patricia Ryan, Robert Tobiansky

**Affiliations:** a Department of Practice and Policy, UCL School of PharmacyLondon, UK; b Alzheimer's Society, Barnet BranchLondon, UK; 3 Memory Service, Springwell Centre, Barnet HospitalLondon, UK

**Keywords:** dementia, family carers, medication

## Abstract

**Objectives:**

Many family carers provide assistance with medicines that is vital for optimal clinical outcomes. Medicines-related tasks are known to contribute to carer burden and stress. This study examined the experiences of family carers when providing medicines-related assistance for a person with dementia, to indicate how services could become more responsive to the specific needs of this group of carers.

**Methods:**

Semi-structured interviews were undertaken with family carers and care-recipients identified though a memory clinic in north London and a local Alzheimer's Society. The interview guide, comprising open questions, was informed by previous studies and consultation with stakeholders. Qualitative procedures involving a framework approach were employed in the analysis.

**Key findings:**

Fourteen interviews with carers and five with care-recipients were conducted. These highlighted the burden and challenges, surrounding medicines-management activities. As well as practical aspects that could be complex, carers were commonly making judgements about the need for and appropriateness of medicines. Although experiences were varied, carers reported difficulties in maintaining supplies, ensuring adherence to regimens and accessing health professionals; and they made some recommendations for service improvements. Carers’ difficulty in obtaining information and advice about medicines was compounded by their desire to allow the care-recipient to retain autonomy over their medicines as long as possible.

**Conclusion:**

This study highlights the distinct needs and problems with regard to medicines-management when caring for a person with dementia. As the prevalence of dementia rises, interventions designed to address these specific aspects of reduce carer-burden should be a priority for health professionals.

## Introduction

The numbers of older people in the UK, as elsewhere, is steadily rising with an increasing reliance on family carers. It is estimated that 23% of the UK population will be older than 65 years in 2035, of which nearly a quarter will be over 85 years.[1] Incidence rates for dementia are also rising. Currently in the UK there are an estimated 800 000 people with dementia, by 2021 this is expected to increase to 1 million. Over two-thirds of people with dementia live in the community. They are supported in their daily living by 670 000 family carers.[1] With these demographic changes and the rising prevalence of dementia, it is anticipated there will be a corresponding rise in the numbers of, and dependence on, family carers.

The UK Government recognises the important role of family carers and their contribution to health care, potentially enabling older people to remain in their own homes for longer.[2] The value of the support of family carers has been estimated at £119 billion pa (significantly more than is spent on the National Health Service (NHS)).[3] Support for family carers has been identified by the UK Department of Health as a policy priority, highlighting the need to improve information and support for family carers to enable them to be effective in their roles.[4],[5] Many family carers are older people and frail themselves.

Assistance with medicines is an integral part of the caring role for many family carers.[3] Older people are major consumers of medicines, and many receive vital assistance from family carers in their use. The range of medicines-related activities that carers can assume is wide,6–8 ranging from occasional assistance, e.g. collecting prescriptions from a surgery or pharmacy, to regular attendance as necessitated by frequent dosing regimens, assisting in the administration of different dosage forms and/or advising on the need for, and use of, various medicines. It is known that all medicines-related activities can present problems for carers. Ensuring that a person has a continuous supply of their medicines can be a challenging task, e.g. different storage sites in the home, pack sizes, formulations and variable need for medicines can all contribute to these difficulties. Older people including those with dementia are likely to have a number of comorbidities requiring a range of different medicines. When dosing regimens are frequent or complex, it can increase the burden especially for carers who have competing demands such as work commitments or children to look after. Problems and concerns are associated with all aspects of carers’ activities when assisting with medicines.7,9–11

When caring for a person with dementia, medicines-related activities can become more complex. Family carers may progressively assume greater responsibilities for all aspects of medicines use Thus, the burden associated with medicines-related activities will often increase. Assistance required from carers will evolve as a consequence of limited memory and understanding, possible lack of insight and emergence of challenging behaviours. A recent study, in Australia, found that carers of people with dementia had distinct needs regarding medication management activities that contributed to carer-burden and stress, often unacknowledged by health professionals.[12] The UK Government in its National Service Framework for Older People (a policy document for service development) acknowledged that at least half of older people may not be taking their medicines as intended and that older people and carers should be more involved in therapy decisions and receive more information on the risks and benefits.[13]

Supporting both care-recipients and carers can present dilemmas for health professionals. There are often no formal channels for imparting information to carers, as services are mindful of preserving confidentiality and autonomy of care-recipients. However, as disease progresses, ways in which ongoing support for carers can be provided so they remain effective in their roles need to be addressed. The provision of care in the context of changing situations and needs of both carers and care-recipients, and the operation of the partnership between them will present challenges for health professionals.[14] The Royal College of General Practitioners has highlighted the need for support for carers within health service provision but acknowledged that ‘the best ideas are still being conceptualised and that they will have to be evaluated’.[15] They also recognise the importance of drawing on carers’ experiences in informing the development of services.

The aims of this study were to examine the scope and range of medicines-related assistance provided by family carers of people with dementia, the problems that arise and to identify how service provision could become more responsive to these needs.

## Methods

This descriptive qualitative study was undertaken in collaboration with the Barnet Memory Clinic (a National Health Service clinic in the northern suburbs of London) and the Barnet branch of the Alzheimer's Society, a voluntary organisation that supports carers and patients with memory problems. Data were collected in face-to-face semi-structured interviews with carers and, when appropriate (able and willing to consent) with care-recipients also.

### Sampling and recruitment

Family carers of, and people with, dementia were recruited through the memory treatment clinics at Barnet Hospital, and the Barnet Branch of the Alzheimer's Society. These two sources enabled the involvement of carers of, and people with, varying degrees of severity of disease as well as people from a wide range of socio-economic and cultural backgrounds.

Carers were eligible if they provided some assistance (however minimal) with a medication for a person with a diagnosis of dementia, were unpaid for the assistance they provided, had at least weekly face-to-face contact with the person they assisted and were the main (informal) carer. The inclusion criteria for care-recipients were: a diagnosis of dementia, living at home and able and willing to consent. The guidance of the Mental Capacity Act: Code of Practice for researchers,[16] was followed whereby potential participants were not excluded on the assumption of incapacity to consent whilst always endeavouring to ensure a voluntary decision.

Letters of invitation (total 85), which included a digital photo of the interviewer, separate information leaflets for carers and care-recipients, and a reply slip were forwarded by mail to potential participants identified by clinic staff and the administrator of the Barnet Branch of the Alzheimer's Society. Carers and care-recipients who indicated their willingness to participate, and were eligible, were contacted by the researcher to arrange a time for a face-to-face interview at their home or another location if preferred. Recruitment continued until data saturation (no new issues were emerging) was achieved.

### Data collection and instruments

The interview schedule for carers was a semi-structured instrument to gather data on medicines-related activities and problems experienced by carers in assisting their care-recipient with their medicines. The domains of the instrument were based on a number of previous studies of carers and medicines,6–8 and in consultation with all members of the research team (health professionals and representatives of care-recipients and carers). Topics included monitoring supplies in the home, liaising with health professionals (hospitals, surgeries and pharmacies), reminders, assistance with administration of different formulations, participating in decisions about the need for medicines, doses and side effects. Carers were asked about help provided and care-recipients about the help that they received. While following this broad framework, interviews were conducted according to principles of qualitative enquiry, and the use of open questions and exploratory prompts to obtain participants’ own accounts, examples of helpful practice or recommendations for service improvements. Personal information was collected including age, sex and relationship of carer to the care-recipient. Informed consent from participants was obtained prior to the commencement of any interview. With the permission of participants, interviews were audio-recorded.

### Data processing and analysis

All interviews were transcribed verbatim. A framework approach to analysis was undertaken[17] involving the development of an initial coding framework guided by responses within each domain of the interview schedule. As analysis proceeded, this was modified and refined using constant comparison techniques, in which all items of data assigned a particular code were appraised for similarities and divergences from those already coded. Computer software (NVivo, QSR International, Burlington, MA, USA) was used to assist in the data management and handling. To ensure the reliability of analytical procedures, all stages of the data processing, coding and analysis involved two members of the research team.

Ethical approval for the study from the NHS Research Ethics Committee of Moorfields and Whittington, London, was obtained prior to the commencement of the data study.

## Results

### The participants

A total of 14 interviews were conducted with carers. In five cases, it was also possible to interview the care-recipient. Fourteen (nine carers and five care-recipients) were conducted at the homes of the participants. Four interviews with carers were held in the clinic and one at a local coffee shop. All participants agreed to audio-recording.

The 14 carers ranged from 45 to 86 years, and the five care-recipients from 81 to 93 years. Eleven of the 14 carers and all five care-recipients were women (see Table 1).

**Table 1:** Characteristics of participants

Participants	Carers	Care-recipients
Women	11	5
Men	3	0
Age-range	45–86 years (11 under 65 years)	81–93 years

The carers included 10 daughters, two sons, one husband and one wife. Five carers lived with the care-recipient. The length of time that carers felt able to leave their care-recipient alone provided an indication of severity of disease. Two carers said they could leave them for much of the day and at night, four could leave them for parts of the day, but not at night. Two carers reported the care-recipient could only be left for a couple of hours. The remaining six would not leave the care-recipient alone.

The numbers of medicines taken by care-recipients ranged from 1 to 15 (mean 7). In addition to medication for dementia (usually donepezil), a wide range of medicines and formulations were used for the management of concomitant conditions, including cardiovascular disease, respiratory problems, osteoporosis, joint pain and mental health problems.

### Interviews with care-recipients

We included care-recipients in the study as we wished to obtain their perspectives on the use of medicines and assistance received. However, in many cases, it was difficult to achieve much meaningful discussion, because of the severity of disease. There were often discrepancies in the information provided by care-recipients and carers, which when verified in other parts of the interviews indicated the more limited knowledge and understanding by the care-recipients.

Care-recipients’ responses were generally brief. Only one of the five care-recipients could comment on the medicines they were taking. However, they all acknowledged that they received assistance, which was generally valued and indicated their carers should be adequately informed.

Well my family need to know all about it, they really do. Because there is no point telling me, so they have to know everything. (Care-recipient 4)

### Interviews with family carers

The interviews with family carers provided detailed descriptions of the scope of assistance provided and the problems experienced. The results are presented according to the seven themes that emerged in the analysis: ordering and collecting from the surgery and/or pharmacy; dosage boxes, reminders and administration; information about medicines; carers’ concerns about the effect of medicines; carers, care-recipients and sharing of information; liaison with health professionals; and suggestions for service developments.

#### Ordering and collecting from the surgery and/or pharmacy

All carers assisted in ordering and/or collecting medicines from the surgery and/or pharmacy, although the pharmacy delivered in four cases. These activities were described in the context of monitoring supplies and arranging timely refills.

… and so I monitor what medications she has and hasn't got left. And when we need more I ring through to the medical practice and ask for a new prescription and then collect them from the pharmacy. (Carer 8)

Most of the carers described the systems they put in place to ensure continuous supplies which, although requiring continual attention, often worked smoothly. However, changes to prescription both added to carer-burden (by creating additional tasks) and stress for carers who wanted to be sure that care-recipients were receiving the right medicines.

The medication was delivered to my mum by the pharmacy. But suddenly they stopped. … I live the other side of London. They did not mention this earlier. So I had to spend another day phoning around to get everything done. I had come not to worry too much if she missed a day of the ramipril or aspirin, because these pills were more preventative. But I didn't want her to miss the Aricept [donepezil]. After that I wrote down in my agenda when to get a new prescription. But it would be so much easier if things would go automatically. (Carer 4)

Monitoring supplies and making timely orders could be a complex task, especially if prescriptions were obtained from more than one source (e.g. hospital clinic and general practitioner (GP)), there were differing ordering procedures, and/or varying lengths of supply for different medicines.

#### Dosage boxes, reminders and administration

Six carers filled dosage boxes (placing tablets in compartments for each day of the week and time of day). In one case a pharmacist did this. For some carers, a dosage box was helpful and reduced carers’ concerns regarding whether the right medicines were being taken.

I make a dosage box every week with all her medicines in, so it is easier to take them, and we are sure she takes the right ones. (Carer 1)

Dosage boxes were also an additional task for carers, increasing the burden of caring and associated anxieties regarding the potential for errors:

There was one day that the agency suddenly phoned up and told me all my mother's pills will have to be in a dosage box. And that had to happen in 48 h, otherwise they would stop giving her the pills. They were trying to protect their carers and I understand that, but they could have given notice a bit earlier. So I had to rush out and buy a little plastic box and move all the pills that the chemist was supplying. And I was as afraid as the carers to make a mistake. It meant I had to make this box every week. (Carer 4)

However, carers may prefer to take responsibility and know that prescriptions were correct:

I don't want the pharmacist to make the box, because they change quite often the medications of my wife, it is much easier for me to make the box. (Carer 5)

Ensuring that care-recipients took their medicines on time was important to many carers. Thirteen (of the 14) carers gave reminders to take medicines. For carers who did not live with the care-recipient, this might be timely phone calls, but it sometimes necessitated regular and frequent visits.

Every afternoon after my work, I go to my mother's house and make sure she takes her medicines and the same thing when she goes to bed. Last 4 years, I've not been away. It's impossible, she needs my help. (Carer 1)

Missed doses as a consequence of reluctance on the part of the care-recipient to take medication, difficulties swallowing or poor memory could be a concern for carers:

Some days he just refused to take them. That happened almost weekly and then we were not able to give the medicines because he did not want to take them. Though sometimes he took them when you waited for a little time. (Carer 2)

She thinks she doesn't need them… It's a shame that she can't remember why she was told to take them. (Carer 9)

#### Information about medicines

Many carers were proactive in seeking out information about medicines. They described reading package information, researching on the Internet, magazines, telephone calls to a doctor and two carers had access to a BNF. For some carers, doctors (GP or memory clinic) were the principal source of information, although insufficient time in consultations to ask questions was also a concern. Information about what, or how much, to take and potential side effects were identified by carers as the most important issues.

Particularly with any new medication, carers wanted information on potential side-effects. Seeking this information was often the impetus for research on the Internet or advice from others. Package inserts were important sources, but interpretation of information and making judgements presented difficulties:

When she lived alone and had to take her blood pressure tablets on her own and I came in the afternoon, the tablet was still in the dosage box and I didn't know what to do. I did not know if it could hurt her to give the tablet at that moment or if I had to wait until the next morning. (Carer 1)

#### Carers concerns about the effect of medicines

Carers were also watchful regarding the effectiveness and in particular potential side effects of medication, often making judgements on the appropriateness of therapy and/or intervening when deemed necessary:

… when he was taking Aricept it had not the right effect on him and it made him worse in his behaviour. Carer 2)

… I was reading the digoxin leaflet lately and it says that on the things digoxin does is confusion and Mum is taking a really high dose of digoxin, so we don't know if we stop that, that will improve her memory. (Carer 12)

She feels terribly tired all the time. I'm not sure if this is a side-effect of the new medicine. (Carer 5)

#### Carers, care-recipients and sharing information

Many carers endeavoured to share information with the care-recipient and wanted to support their involvement in decisions about their medicines. However, also, care-recipients wishing to remain autonomous over their medicines was problematic for some carers:

It is really difficult. Mum… forgets. What I've done is sort of sit down and explain things, and her own family doctor has tried to explain things. I want Mum to know what is going on. She is very independent.(Carer 11)

I used to give her information, but now she forgets all. (Carer 3)

..my mother won't let me [be involved in decisions] because she wants to be independent. (Carer 10)

Because carers might not be present in a consultation, they were often not informed of medication and regimens prescribed. This was especially difficult when there were any changes to a prescription.

… it is wise to invite a member of the family so if that person forgets there is someone else, hopefully, to remember what information is given. (Carer 8)

the doctor doubled the dosage without even telling us, we only found it out by collecting the tablets at the pharmacy. (Carer 12)

#### Liaison with health professionals: support for carers

Many carers described positive experiences regarding the support they received from health professionals, especially in the clinics they attended. However, problems were experienced in contacting health professionals, especially on behalf of care-recipients, and in particular in accessing the care-recipient's GP:

I have no good contact with my mother's GP. I cannot reach her normally, only by receptionist or by post. I would prefer to contact by phone or email, but for a strange reason that is not possible. And I really want to discuss some things with her… Carers should be involved always and as much as possible. Because they are the ones who will remember the information. (Carer 3)

However, when carers were present, they sometimes described how the consultation may then exclude the care-recipient. As discussed above, carers endeavoured to share information with care-recipients; they also believed care-recipients should be involved in discussions:

… and sometimes the doctor is talking to me about my dad when he is sitting next to me, but they should talk to him because he can answer the questions better than I can. I think they have to know he is not that bad. (Carer 7)

Our GP was talking only to me as if she was not there. My mother thought he was saying she was mad because he was not talking to her. I was so angry. (Carer 6)

… they should attend the discussion as everybody else. They will forget it afterwards but I think it is insulting not to talk to the patients.(Carer 8)

### Suggestions by carers for service developments

A number of suggestions were made by carers for service developments. These related to the provision of information; access to, and communication with, GPs; and pharmacy services (see Table 2).

**Table 2:** Suggestions by carers for service developments

Information Provision of information in the second consultation: avoiding too much at initial diagnosis but ensuring it provided at any early stage. Providing information about what medicines are for, side effects, what to look out for Routine communication of important information to the carer, as well as the care-recipient, especially if the carer cannot be at consultation, e.g. when medicines or regimens are changed.
GPs: access and communication GPs to be more accessible for carers, e.g. recognition by surgery staff of role of carers and being prepared to talk to them. Timing of consultations that accommodate carer's competing commitments, especially for carers who work Clear and accessible systems for contact by telephone or e-mail More training for GPs in communicating with people with memory problems Simplified systems for ordering prescriptions, e.g. computers that recognise when new prescription is due.
Pharmacists and potential roles Pharmacists could assist in ensuring medicines refills are timely Pharmacists can be good source of advice, but consistency between pharmacies in quality of services needs to be addressed.

## Discussion

The findings of this study illustrate the essential help with medicines that family carers of people with dementia provide and confirms how medicines-related activities are a conspicuous and potentially stressful part of the caring role.

This study was limited in that it involved just a small number of carers from one part of London. Although the needs and perspectives of family carers in assisting with medicines may be replicated elsewhere, differences, e.g. in service provision, may affect carers’ experiences.

Making sure care-recipients received the right medicines at the right time was a priority for carers. Carers described how they had to understand regimens and ordering procedures, and take responsibility for monitoring requirements. The continual checking required could be burdensome and when, inadvertently supplies were low, securing continuous supplies could be stressful. Carers also made their own assessments regarding the suitability of medicines with regard to perceived benefits and (potential or actual) adverse effects. In these tasks, carers often did not feel adequately informed or experienced difficulties in interpreting and applying medicines information. They described difficulties in accessing health professionals for advice, which might assist them in making these judgements.

Carers’ problems in assisting these care-recipients with medicines could also be compounded by a lack of understanding and challenging behaviours of the person they were assisting. Sometimes differing views of carers and care-recipients regarding the need for medicines could present dilemmas for carers, who despite witnessing symptoms of disease progression, generally wished to encourage patient autonomy in decisions regarding the use of medicines. Carers were generally strong advocates of patient autonomy and worked in their best interests. Accordingly, care-recipients interviewed in this study, though limited in the perspectives they could provide, acknowledged their dependence on their carers.

Carers’ experiences of communication and support from health professionals was hugely variable. While some carers identified key health professionals and clinic staff who were understanding and helpful, some carers identified areas to be addressed. Carers were keen for care-recipients to be informed, involved in decisions and to retain control over their medicines as much as possible. However, consultations that focus on a care-recipient's needs should also address those of carers.

Some carers also expressed dissatisfaction with the balance and focus of consultations involving a health professional, patient and carer. This might be insufficient attention to the perspectives of either the care-recipient or the carer. As disease progresses many carers, while wanting the care-recipient to remain central, were frustrated at the difficulties of ensuring their need for information and concerns were addressed. To ensure that carers are able to perform their roles effectively, there is a balance to be achieved between preserving care-recipient's autonomy and independence while ensuring a process in which carers are involved, informed and supported in their roles.

Carers also experienced difficulties in being able to contact health professionals themselves for information. While some consultations were helpful, outside these, formal channels of communication for carers were not described. Issues of confidentiality and patient autonomy may present barriers. A particular problem for carers was that they would not necessarily be informed of changes to prescriptions or regimens. This difficulty has been identified in other studies[9],[12] and reflects poor systems for communication between primary and secondary care in the UK as well as with family carers.[13],[18]

While other studies have identified medication-related roles of family carers,6–8 assisting a person with dementia presents additional issues that need to be taken into account in designing services.[15] However, many older people will have many differing needs and some who do not have a diagnosis of dementia may experience memory problems that will have implications for carers. Carers in this study made a number of practical suggestions: improved processes and practices regarding communication with GPs, systems to improve transfer and timing of medicines-related information; and possible roles of pharmacy services in assisting in timely refills and information about medicines. In recent years there have been initiatives in the UK, to support optimal use of medicines by care-recipients living in their own homes. These include Medicines Use Review, a widely used service delivered by community pharmacists[19] for which there i6s some evidence that they have a positive effect on adherence, medicines use and clinical outcomes although more research is required to be certain of these effects.[20] However, at present, these do not formally include and separately address the needs family carers.

The study highlights the need for the provision of training and support for family carers that cover a wide range of factors, including specific attention to medication. The Barnet Alzheimer's Society, who collaborated in this study, now have an information and support officer whose role could include such interventions.

However, the development of interventions to reduce associated carer-burden and enable carers to be effective needs to be a priority for health professionals, and in their evaluation, carer-burden associated with medicines-management activities is an important outcome measure. Instruments currently available[21] do not specifically address the added dimensions and challenges of caring for a person with dementia. The activities, problems and needs of carers as identified in this study could provide a starting point for the development of a relevant and sensitive outcome measure.

## Declarations

### Conflict of interest

The Author(s) declare(s) that they have no conflicts of interest to disclose.

### Funding

This research received no specific grant from any funding agency in the public, commercial or not-for profit sectors.
